# Genetic Variations in IL-1β, TNF-α, and TGF-β Associated with the Severity of Chronic Cervical Spondylitis in Patients

**DOI:** 10.3390/cells12121594

**Published:** 2023-06-09

**Authors:** Shashi Ranjan Mani Yadav, Bela Goyal, Garima Mamgain, Ashish Kothari, Sandeep Kumar, Sarama Saha, Manisha Naithani, Anissa Atif Mirza, Raj Kumar, Rajnish Arora

**Affiliations:** 1 Department of Biochemistry, All India Institute of Medical Sciences, Rishikesh 249203, India; 2 Department of Medical Oncology/Hematology, All India Institute of Medical Sciences, Rishikesh 249203, India; 3 Department of Microbiology, All India Institute of Medical Sciences, Rishikesh 249203, India; 4 Department of Medicine, OUHSC, Oklahoma City, OK 73104, USA; 5 Department of Neurosurgery, All India Institute of Medical Sciences, Rishikesh 249203, India

**Keywords:** chronic cervical spondylitis, polymorphisms, pro-inflammatory, anti-inflammatory, PCR-RFLP

## Abstract

Chronic cervical spondylitis (CCS), a degenerative disorder of the spine, is known for causing disability among old and young people. Single-nucleotide polymorphisms (SNPs) in various cytokine genes have demonstrated an impactful association with several inflammatory disorders. In the present study, we have investigated the SNPs and allelic distribution of the three most prevalent cytokines genes, IL-1β (-511C/T), TNF-α (-308G/A), and TGF-β (-509C/T), along with serum levels of these cytokines in 252 subjects. SNPs were analyzed using the polymerase chain reaction-restriction fragment length polymorphism (PCR-RFLP), and digested fragments were separated and visualized using agarose gel electrophoresis and Native Polyacrylamide gel electrophoresis (PAGE). The serum cytokine levels were analyzed with a flow cytometer using a customized multiplex bead-based assay. It was observed that these SNPs did not reflect the susceptibility to CCS but were associated with susceptibility to CCS. We found a significant association between the C/C and G/G genotypes and the C and G alleles of IL-1β and TNF-α, respectively, suggesting a lower risk of CCS. The frequency distribution of risk alleles (-511T) and (-308A) were simultaneously higher in CCS compared to the control, reflecting the susceptibility to CCS. TGF-β showed a significant association with disease susceptibility, along with a significant correlation between age and the chronicity of CCS. The serum cytokine levels were significantly different in CCS and controls.

## 1. Introduction

Chronic cervical spondylosis (CCS) is a common chronic and progressive degenerative disorder of the cervical spine that adversely affects postural health. It is reported in demographic studies that about 80–90% of people showed disc degeneration on magnetic resonance imaging (MRI) by the age of 50 years [1]. Although cervical spondylosis is widely believed to be a disease of old age, with age-related degeneration being the most important factor, it is now widely reported in younger age groups as well. It is widely believed that the natural aging process is the primary cause of degeneration of the osseocartilaginous elements of the spine; however, this theory is not supported by any of specific evidence [1,2]. Several environmental risk factors such as body mass index (BMI), physical workload, smoking, and occupation have also been imposed on intervertebral disc degeneration. However, genetic factors are implicated more than environmental factors for children 11–16 years old [3]. The functional outcomes of genetic variations could be observed in the phenotypic characterization of disease [4]. The study of polymorphism is sought, as it may predict earlier risk for degeneration because it is ubiquitous in the genome [5]. Studies have been conducted associating gene polymorphisms in deciphering disease status and predicting susceptibility groups [4]. According to the previous literature, disc degeneration is associated with polymorphisms in several genes, such as the Vitamin D Receptor (VDR) and cytokines, which suggests that it is a multi-genetic condition [6,7]. Cytokines are pleiotropic and can act either as proinflammatory or anti-inflammatory depending on their biological properties [8]. Cytokine genes are highly polymorphic with their receptors [4]. Inflammatory cytokine polymorphisms have been studied for susceptibility to other degenerative conditions such as rheumatoid arthritis, ankylosing spondylitis, etc. [9,10,11]. IL-1β, TNF-α, and TGF-β are the most commonly studied cytokines among the various inflammatory cytokines. Polymorphism in the genes coding for IL-1β has shown a significant relation with the progression of the disease in the degeneration of the cervical and lumber [12,13]. A recent meta-analysis showed an association of IL 1 polymorphism with the risk of IVDD and RA [14,15]. Amongst various IL-1 polymorphisms, IL-1β-511C/T promoter has shown promising susceptibility in the progression of inflammatory disorders such as RA and SLE [15,16] and juvenile idiopathic arthritis as well [17]; however, evidence on the association with CCS is still lacking.

Tumor necrosis factor alpha (TNF-α) adversely mediates bone degradation by facilitating matrix degradation, which exacerbates cervical spondylosis [13]. One study shows that the use of anti-TNF-α antibody and TNF-α receptor antagonist has improved the symptomatic ankylosing spondylitis patients [18]. Previous literature shows an increased amount of TNF-α mRNA and higher serum levels of TNF-α to be associated with inflammation as well as the progression of disc degeneration [13,19]. A substitution of G- to -A at promoter region -308 of the TNF-α gene has been extensively studied for its important role in inflammatory disorders such as ankylosing spondylosis and rheumatoid arthritis [18,20]. Such genetic variants have been reported to alter the production of cytokine levels [21]. Hence, it is possible that genetic variation in the promoter region of TNF-α (-308G/A) could play a significant role in disc degeneration, but there is a lack of sufficient evidence to support the mechanism of pathogenesis, especially in CCS.

Transforming growth factor-β (TGF-β), an anti-inflammatory cytokine has been found to play a protective role in intervertebral disc degradation either by abnormal bone remodeling or by improving proteoglycan matrix deterioration [9], although the putative mechanism for it remains debatable. Polymorphism in the region of the TGF-β gene, especially at promoter -509C/T, has been associated with disc degradation and the disease process. However, most of the previous studies have included IVDD associated with the lumbar spine. IVDD, as one of the components of CCS, is imperative to study the association of these polymorphisms with susceptibility to CCS as well.

The role of ethnicity in variable susceptibility and association of disorders is well known. A recently published meta-analysis on a very closely linked ethnic population showed a significant influence of ethnicity on the association of polymorphism with disease. They reported a significant association of TNF-α (-309A/G) in osteoarthritis (OA) patients from South India, whereas there was a non-significant association in patients from North India [16]. Owing to the variability in genotypic–phenotypic relationships in different ethnic population groups and North Indians being unique due to the difference in environmental, ethnicity, and lifestyle-related factors, it is important to study the genetic markers the for susceptibility to CCS, which is highly prevalent and disabling in the North Indian population.

As there is a lack of authentic research literature, there is an immense need to study the association of cytokines with susceptibility to CCS, so we aimed for a case–control study to explore the association of the proinflammatory genes IL-1β and TNF-α and anti-inflammatory gene TGF-β with CCS in a North Indian Population.

## 2. Materials and Methods

The Institutional Ethical Committee of All India Institute of Medical Sciences (AIIMS) Rishikesh approved the present study for ethical clearance (AIIMS/IEC/19/718). A total of 252 subjects were enrolled, including 126 adult CCS cases from Neurosurgery OPD and a similar number of age-matched (male/female) healthy adult individuals as control at AIIMS Rishikesh, India, from 2018 to 2021. Well-described written informed consent was obtained from all participants.

The diagnosis of CCS was made based on thorough clinical evaluation (clinical history and detailed examination) and radiological (X-ray/CT scan/MRI) findings. The subjects were included as CCS patients if they experienced clinical symptoms, including cervical pain generated by movement, referred pain which radiated to shoulder blades and upper limbs, cervical stiffness, and retro-orbital as well as temporal pain (C1 to C2) for more than three months’ duration or a known case of myelopathy or radiculopathy [22]. Patients with any history of infection or inflammatory diseases such as RA, ankylosing spondylitis (AS), osteomyelitis, polymyalgia rheumatic, Paget’s disease, and osteoporosis, along with bone-degenerative conditions, including non-specific neck pain lesions, mechanical lesions, psychogenic neck pain and many others besides CCS, were excluded [22]. Controls were free of any degenerative or non-degenerative and inflammatory or non-inflammatory diseases and lacked any positive family history for them. This was ascertained by analyzing CRP and ESR for the controls. All CCS patients and control were North Indian population in origin and non-consanguineous.

### 2.1. Measures of Chronicity

Chronicity has been considered based on the duration of pain experienced by CCS patients, and the period should be more than 3 months of chronic pain experienced [22]. All CCS cases were grouped into two categories of chronicity for convenient analysis, i.e., >3 months and >1 year.

#### 2.1.1. Erythrocyte Sedimentation Rate (ESR) and C-Reactive Peptide (CRP) Measurement

Erythrocyte Sedimentation Rate (ESR) and serum C—reactive protein (CRP) levels also confirmed the inflammatory status of control. The samples collected from the control and patients were measured for ESR by the Westergren method following the procedure described previously [23]. The CRP levels in the serum of controls were analyzed by using an immuno-turbidimetric assay kit (CRP Turbilatex, Beacon, NY, USA) using an AU480 analyzer (Beckman Coulter, Brea, CA, USA).

#### 2.1.2. Genotypic Analysis

A total of 5 mL of blood was collected in EDTA vials and non-additive vials from respective patients. The EDTA collected samples were either processed immediately or stored at 2 to 8 °C until further use for DNA isolation from WBCs as per the need of experiments. The serum was separated from the non-additive collected blood after clot formation at room temperature and stored at −80 °C for further analysis.

#### 2.1.3. DNA Isolation

Genomic DNA was isolated from peripheral blood with the QIAamp kits (QIAGEN, Hilden, Germany) following the manufacturer’s instructions. The isolated DNA was quantified on TECAN Multi-Mode Reader, USA, by taking the optical density at 260 nm. The purity of DNA was checked by calculating the ratio of absorbance at 260 to 280 nm. The agarose gel electrophoresis (0.8% *w*/*v*) was used to screen the integrity of genomic DNA. Further, the genomic DNA was used to amplify the desired gene segment using a polymerase chain reaction by employing gene-specific primers.

#### 2.1.4. Selection of SNPs and SNP Genotyping

Three different SNPs were selected based on previous studies that have reported their association with other degenerative and inflammatory disorders: IL-1β gene (promoter-511C/T) [24], TNF-α gene (-308G/A) [19], and TGF-β gene (-509C/T) [25].

Genotyping of IL-1β-511C/T, TNF-α-308C/T, and TGF-β-509C/T was performed by employing PCR- RFLP followed by non-denaturing agarose gel electrophoresis and Native-PAGE for smaller fragments. Forward and reverse primers were designed according to previously reported studies [19,24,25], and amplification was carried out in the lab (Master cycler Gradient Thermocycler, Eppendorf, Germany). The list of primers and respective restriction enzymes is provided in Table 1. In brief, the PCR amplification reaction was performed in 20 µL of assay mixture, containing genomic DNA (100 ng) and PCR master mix (10 µL), which is composed of Taq DNA polymerase (0.05 U/µL), MgCl2 (4 mM), reaction buffer, dNTPs (0.4 mM each) (Thermo Scientific, Waltham, MA, USA), and forward and reverse primers (25 pM each) (IDT, Coralville, IA, USA). The PCR reaction mixture was initially denatured at 95 °C for 3 min, followed by 35 cycles of denaturation at 95 °C for 30 s. The annealing temperatures for IL-1β, TNF-α, and TGF-β were 45 °C, 60 °C, and 62 °C, respectively, for a period of 30 s each. The extension temperature for all genes was 72 °C with a varying period of 20 s for IL-1β, 10 s for TNF-α, and 45 s for TGF-β. An additional final extension was employed for all three genes at 72 °C for 5 min.

The PCR amplicon of IL-1β was digested with AvaI restriction endonuclease (Thermo Scientific, Waltham, MA, USA) at 37 °C for 60 min. The sizes of digested fragments were 190 bp and 115 bp in the wild type, 305 bp in the homozygous variant, and 190 bp, 115 bp, and 305 bp in the heterozygous variant (Supplementary Figure S1). PCR amplicon of TNF-α was digested with NcoI at 37 °C for 1 h. The sizes of digested fragments were 87 bp and 20 bp in the wild-type genotype, 107 bp in the homozygous genotype, and 107 bp, 87 bp, and 20 bp in the heterozygous genotype Supplementary Figure S2). The PCR amplicon of TGF-β was digested for one hour with Eco81I at 37 °C. The size of digested fragments was 81 bp in the wild genotype, 42 bp and 39 bp in the homozygous genotype, and 42 bp, 39 bp, and 81 bp in the heterozygous genotype (Supplementary Figure S3). All the restriction endonuclease digestion was followed by deactivation at 70 °C for 20 min.

The PCR amplicon and their digested fragments of IL-1β and TNF-α were separated on 2% agarose gel (*w*/*v*) electrophoresis, while that of TGF-β was separated by running 12% native PAGE (Bio-Rad, Hercules, CA, USA).

The agarose gels and PAGE gels were stained with ethidium bromide, and images were captured by a gel documentation system coupled with a UV trans-illuminator (GelDoc, SynGene, Iselin, NJ, USA).

### 2.2. Measurement of Serum Cytokines by Flow Cytometer

The sera collected in non-additive vials of CCS and controls were stored at −80 °C in different 200 uL aliquots to avoid repeated freeze and thaw cycles. The serum aliquot was taken, thawed, and promptly stained with antibodies, followed by quantification using a LEGENDplexTM Custom Human 6-plex Panel based on multiplex bead-based assay (LEGENDplex™, Biolegend Inc., San Diego, CA, USA). The panel was customized to analyze 6 inflammatory cytokines, namely IL-1β, IFN-γ, TNF-α, TGF-β, IL-1RA, and IL-10. The procedure for staining was as per the suggestion in the manufacturer’s instructions. The analysis with flow cytometry was performed employing BD FACS Canto II (Becton Dickinson Biosciences, San Jose, CA, USA). The raw data acquired (x.fcs) were interpreted using LEGENDplexTM Data Analysis Software V-8.0. The LEGENDplex chip platform opted for the detection of signal value generated. The standard curve was the basis for the calculation of serum concentration. The concentration range for IL-1β and TNF-α cytokine was 0–10,000 pg/mL, while that for TGF-β was 0–20,000 pg/mL. The sensitivity of detection was achieved at 5–50 pg/mL, and the coefficients of variation (CVs) of the standard for cytokines IL-1β, TNF-α, and TGF-β was achieved as the medians (ranges) of 3.9 (1.01–9.7), 5.9 (1.6–33.3), and 5.45 (0–14.4), respectively.

#### 2.2.1. DNA Sequencing

After successful PCR, we randomly selected 10% controls and CCS out of the total study population. The sequencing was performed with reverse and forward primers by using the Applied Biosystems 3730xL Dx Genetic Analyzer (Applied Biosystems, Foster City, CA, USA), and the BigDye^®^ Terminator v3.1 cycle sequencing kit (Invitrogen, Thermo Fisher Scientific, Waltham, MA, USA) was used for the sequence analysis.

#### 2.2.2. Statistical Analysis

The normally distributed data were presented in the mean ±  SD, and the median with interquartile range (IQR) was used for non-parametric data. The Chi-Square test for categorical and Mann–Whitney U *t*-test data for quantitative were used to compare the basic demographic data between groups. Descriptive statistics were used to calculate frequencies of genotypes and alleles of SNPs as well as the cytokines level of respective genotypes. A Chi-Square test or Fisher’s exact test was used to compare allele and genotype frequencies among groups and the Hardy–Weinberg equilibrium of genotype distribution.

The Odds Ratio (OR) with 95% confidence intervals (CIs) was calculated to assess the relative risk conferred by a particular allele and genotype. To provide a separate OR for each genotype, the most common genotype was considered as the reference group. Linear regression (R^2^) was used to show the association among the genes. The genotypic and allelic frequency distribution was analyzed with GraphPad Prism Software version 5.1 (GraphPad, La Jolla, CA, USA). All other statistical analyses were performed with the SPSS statistical software (version 25.0) and *p*-values < 0.05 were considered statistically significant. The *p*-values were adjusted for multiple comparisons using the Bonferroni method.

## 3. Results

### 3.1. Study Population and General Characterization

Among the total CCS subjects enrolled, 58 were males (46.03%), and among healthy control, 73 were males (57.94%), while the rest were females. The mean age (years) with SD was 47 ± 10.89 for CCS and 45 ± 11.32 for controls. The average height of CCS subjects enrolled was 157.87 ± 7.99 cm, while in the controls it was 156.01 ± 8.25 cm. The frequency of smokers among CCS was 29 (23%), while in the control was 31 (24.6%) (Table 2). These data had no significant association between CCS and control. The sample size estimation and the statistical power calculation were conducted based on the allele frequency of the variants. The study sample size is sufficient to reach 80% power at a significant level of 0.05 when considering the medium effect size of 0.3.

Inflammatory markers such as ESR were found to be significantly different among CCS and controls, with means ± SD being 40.056 ± 12.53 mm/h and 13.82 ± 4.75 mm/h, respectively. Serum CRP levels were analyzed only for controls and were found to be within the normal range (mean ± SD = 1.68 ± 1.69 mg/L). These data ensured the absence of any systemic or local inflammation in controls for inclusion in the study.

Frequencies of TGF-β SNPs in both CCS and control, as well as the distribution of TNF-α in CCS, deviated from Hardy–Weinberg equilibrium, while polymorphism frequencies of genotypes IL-1β in CCS and Control and TNF-α in Control were consistent with the equilibrium.

### 3.2. Allele and Genotype Frequencies of Cytokines

The genotypic and allelic variations of IL-1β (-511C/T), TNF-α (-308G/A), and TGF-β (-509C/T) were analyzed in CCS patients compared to controls to uncover any associations with CCS. The genotypic and allelic frequencies of IL-1β were not significantly associated with CCS, whereas there was a borderline significant difference in genotype distribution of CC over TT of IL-1β (*p* < 0.049). The frequency of the presence of polymorphic C-allele of IL-1β was relatively higher in control (55%) than in CCS (45%) with *p* < 0.05. Further statistical analysis showed that the C-allele might have a protective role in the progression of CCS (OR = 0.68 and 95% confidence interval (CI): 0.48–0.97). These associations further disappeared and were found to be non-significant when applying a correction for multiple analyses (*p* > 0.05).

We found a significant difference in CCS and control concerning the distribution of genotypes of TNF-α (*p* < 0.0001) even after adjusting *p*-values for multiple comparisons. The frequency of G-allele of TNF-α was present in an increased number in control (94%) than in CCS (60%), which was highly significant (*p* < 0.0001). Regression analysis of the allele showed that it might also have a protective role in CCS development (OR = 0.87 and 95% CI: 0.05–0.16).

There were significant differences in genotypic distribution among CCS and control (*p* < 0.005). However, there was no significant association between the alleles of TGF-β in CCS and control (Table 3).

When different age groups of subjects in CCS and Control were compared, the genotypes and alleles of IL-1β, TNF-α, and TGF-β were found to have comparable results (*p* > 0.05) (Table 4).

Further, when these genotypes and alleles of IL-1β, TNF-α, and TGF-β and their effects on chronicity were taken into consideration, the results were comparable and showed no significant association among CCS (*p* > 0.05) (Table 5). In the comparison of smokers and non-smokers among CCS and control, there was no significant relation between genotypes or alleles of IL-1β, TNF-α, and TGF-β among subjects of either CCS or control (*p* > 0.05) (Table 6). On further analyzing smokers in CCS and controls, the results were found to be inconclusive in comparing only smokers of CCS and Controls with the genotypes and their alleles, which were similar to non-smokers (Table 7).

### 3.3. Association of Serum Cytokines with CCS and Control

The serum cytokine levels were found to be significantly higher on comparing with CCS and Controls (*p* < 0.001). The serum levels of TNF-α and TGF-β were significantly different from CCS and Controls (*p* < 0.05 and *p* < 0.001), while that of IL-1β was found to be comparable (*p* > 0.05). We also compared serum cytokine levels with different genotypes and did not find significant associations among genotypes of IL-1β between CCS and control (*p* > 0.05), while a prominent association of significance was observed in that of TNF-α and TGF-β between CCS and control (*p* < 0.05) (Table 8).

On subsequent analysis of serum cytokines in association with chronicity, smoking, and different age groups, we did not find any statistically significant difference between CCS and control (*p* > 0.05). However, there were marked differences in levels of cytokines in severe chronic (>1 year) to chronic (>3 months) and in older (>50 years) to younger (<50 years) cases of CCS than that in Controls (Table 9 and Table 10). Surprisingly, a different trend was observed in smokers to non-smokers, where elevated cytokine levels were found in non-smokers with CCS and smokers of controls. In contrast, cytokine levels among smokers and non-smokers with CCS were comparatively higher than that of controls (Table 11). The arbitrary result could be possible due to the uneven distribution of study subjects.

### 3.4. Pearson Correlation Study of Variables

For the correlation analysis of genotypic variables among CCS and controls, the age of CCS was significantly and positively correlated with chronicity (*p* < 0.005) and was negatively correlated with most variables. The ESR in CCS was negatively correlated and significantly correlated with TGF-β in CCS (*p* < 0.05). Genotypes of IL1-β in CCS were positively correlated with genotypes of TNF-α in CCS and controls simultaneously (*p* < 0.05), while it showed a negative correlation with other variables. TGF-β in CCS and controls was negatively correlated with all the variables compared. (Table 12).

**Figure 1:**
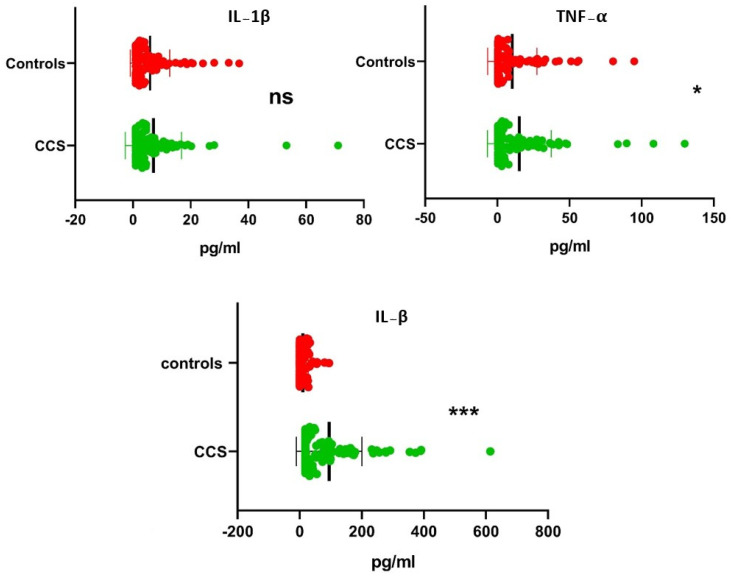
Correlation of serum cytokine levels using Mann–Whitney U–test among CCS and controls; CCS = Chronic Cervical Spondylitis; IL-1β = Serum Interleukin–1β; TNF-α = Serum Tumor Necrosis Factor-α; TGF–β = Serum Transforming Growth Factor–β; ns = non-significant; * levels of significance (* *p* < 0.05; *** *p* < 0.001).

The cytokine levels were studied for correlation among different cytokines and their genotypes in CCS and control, along with chronicity. Although there was no significant association among the variables (>0.05), the level of cytokine IL-1β among controls was found to be significantly correlated with genotypes in CCS of TNF-α (*p* < 0.05) (Table 13, Figure 1).

## 4. Discussion

CCS is multifactorial disorder, with environmental factors such as aging and lifestyle, as well as genetic factors, playing a pivotal role in its pathogenesis as studied through previous literature [26]. 

Polymorphism in genes and their association with the progression of clinical conditions provide great insight into the molecular as well as cellular pathogenesis of these diseases. Genome-wide association studies have evidenced the association of genetic variations at the transcriptional level with the severity of diseases, as observed in patients with neck and shoulder pain [27].

Studies on genetic variations in cytokines have proposed the association of genotypic and allelic variants with susceptibility and severity of various acute and chronic autoimmune disorders [9,28,29,30].

Since immune-inflammatory processes are said to contribute to the degeneration of the spine, it has been observed that alteration in genetic basis may influence spinal trauma, including unnoticed subclinical disc desiccation. The upregulation of proinflammatory cytokines such as IL-1β, TNF-α, and IL-6 have been shown to be associated with diminished matrix-producing cells, leading to low production of hydrophilic proteoglycans, thereby promoting gradual desiccation of the disc [1] in the experimental setup. The genetic polymorphism in these cytokines has been observed to influence the production and release of cytokines, cause imbalance among disease-associated cytokines affecting the susceptibility to inflammatory disorders and their degree of severity in spondylo-arthropathies such as RA, SLE, AS, and Bullous pemphigoid [9,24,28,31].

Inflammatory cytokines have also been studied in CCS, where increased levels of inflammatory cytokines in the sera of CCS patients are associated with the severity of the disease. However, genetic studies on CCS are limited. To the best of our knowledge, this study is the first to investigate the associations of alleles and genotypes of proinflammatory and anti–inflammatory susceptible genes (IL-1β, TNF-α, and TGF-β) with the risk of CCS.

In the present study, in the case of IL-1β, the genotype TT was more frequent in CCS, but no significant association was observed with the disease. Although CC was significantly associated with the disease, the significance disappeared on evaluation with adjustment for multiple analyses.

At the allelic level, the C-allele could resist CCS progression, which appeared to be significantly more frequent in the control than in CCS. T-allele of IL-1β could be a risk allele for CCS progression, as it was more predominant in CCS than in the control. Additionally, there was no significant effect of either of these genotypes or alleles on the serum IL-1β level (*p* > 0.05). Since no studies are available for comparison in CCS, the literature was searched for corroboration with other degenerative and autoimmune disorders. These results are in concurrence with a study conducted by JF Carmago et al. in Colombian patients with RA [32] and SLE, and Mohd Jahid et al. in the North Indian population with RA [24], wherein the C allele was found to be protective and the T allele was found to be associated with disease susceptibility in SLE and RA. However, a study by Lagha et al. showed contradictory results, with C allele being associated with disease susceptibility in RA in the Tunisian population. [33]. Similar findings were reported in the population of Chinese Han with AS [34] and Darwish et al. in Egyptian patients with RA [35]. Besides these documented reports, the C-allele and CC genotype frequencies of IL-1β were observed with the risk of developing Graves’ disease in one study, along with another study of gastric carcinoma; both studies were conducted in the Kashmiri population of North India [36,37]. The plausible reason for these discrepancies could be the genetic heterogeneity in different populations [38], or it could be due to the inhibition of transcription factors by the IL-1β-511 CC genotype to bind promoter regions leading to suppression of gene expression, unlike the IL-1β-511 TT genotype, which positively ameliorates gene expression and exacerbates disc degeneration [24].

In the case of TNF-α, we found that all the three genotypes, i.e., AA/AG/GG, were significantly associated with CCS as compared to controls (*p* < 0.0005). The heterozygous variant A/G was found to be associated with an increased risk of severity of CCS (OR = 4.08; *p* > 0.05). Likewise, we found significantly higher serum TNF-α levels in all the genotypes of CCS as compared to controls (*p* < 0.01), while at the allelic level, G-allele had a highly significant association with CCS over A-allele in comparison with control (OR = 0.87; *p* < 0.0005) and thus could play a role in the protection against the susceptibility to CCS. However, the frequency of the A-allele was predominant in CCS as compared to the control, suggesting its positive contribution to the progression of CCS. This is in accordance with the study conducted in the north Indian population with a similar ethnicity to that in our study by Das S et al., in the disease RA [39], and Raafat et al. reported similar results in the Egyptian population with OA [40]. However, some studies reported a significant association between the GG genotype and the G-allele, showing their distinct role in the severity of RA cases [41,42], while others did not find any association regarding the association of 308G/A variants with inflammatory disorders [43,44]. The conflicting results in different studies in different populations could be explained by genetic heterogeneity [38]. Additionally, TNF-α (308G/A) lies in the regulatory region of TNF-α and is believed to play an important role in the transcription since it may be possible that TNF-α (-308A) allele could manipulate the regulation of gene transcription, causing increased mRNA expression, finally regulating protein production [39,45,46]. The genetic transition of Guanine to Arginine in TNF-α-308G/A has been associated with several inflammatory disorders; however, the associations are quite controversial and depend on ethnicity.

In TGF-β (-509C/T), there was a significant association of all the genotypes with CCS as compared to control assuming frequencies of TT and CC genotypes dominating CCS progression (*p* < 0.005). We also found higher serum TGF-β levels in all the genotypes of CCS as compared to controls (*p* < 0.01). Similar levels of significance were observed between genotypes of TGF-β and their serum levels. However, we did not find any significant association of either of the genotypes or alleles independently contributing to the susceptibility to CCS, except the CC genotype, which could play a prominent role in the severity of CCS (OR = 8.13; *p* > 0.05). This finding is in concurrence with the result of a recently published meta-analysis exploring associations of TGF-β (-509C/T) genotypes in RA patients [28]. However, Iftekhari H et al. did not find a significant contribution of genotypes in their subjects, but the frequency of genotypes CC and TT was higher in CCS than in the control [47]. However, Min-yu-tu et al. showed that the CC-genotype of TGF-β could be protective in the progression of osteoporosis [48]. Liu C et al. showed different findings from ours, where they found TT genotype and T-allele to be significantly associated with the susceptibility to OA, as other studies reported previously [30,49,50,51], although some studies did not find any association [52]. The position of TGF-β (-509C/T) is in the negative regulatory region of the TGF-β1 promotor. The -509C allele suppresses transcription factor (Activated protein 1) AP1, which simultaneously lowers TGF-β1 promoter activity, resulting in decreased protein production [53]. The difference in outcomes from our study and others could be due to diverse ethnicity and environmental factors. This can again be explained by genetic heterogeneity [38].

Wen analyzing different clinic-demographic and serum protein levels, we observed a significant positive correlation between age and chronicity of the disease (Pc = 0.3; *p* < 0.005), suggesting that age could affect CCS progression [47,54]. Similarly, a positive correlation was also observed between serum IL-1β and serum TNF-α levels. This explains the proinflammatory nature of both cytokines, whereas a negative correlation was observed between IL-1β and TNF-α, with TGF-β indicating the anti-inflammatory nature of TGF-β. The correlations align with findings already documented. Although previous studies reported that smoking could affect cervical degeneration, the present study found inconclusive results, and hence it may not be considered an environmental modifier. This could be possible due to large differences in population size considered for the comparison [55,56].

Since it has been shown in a series of studies that IL-1β and TNF-α risk allele has been associated with the risk of inflammatory disorder, our findings have provided comparable evidence. The effect of genetic transitions in the different age categories, chronicity, and smoking habits of the included subjects have not been explored previously with CCS. However, we did not find any significant association among these factors, but the distribution of genotypes and allele frequency between CCS and control with different age categories, chronicity, and smoking habits was of serious concern.

**Figure 2:**
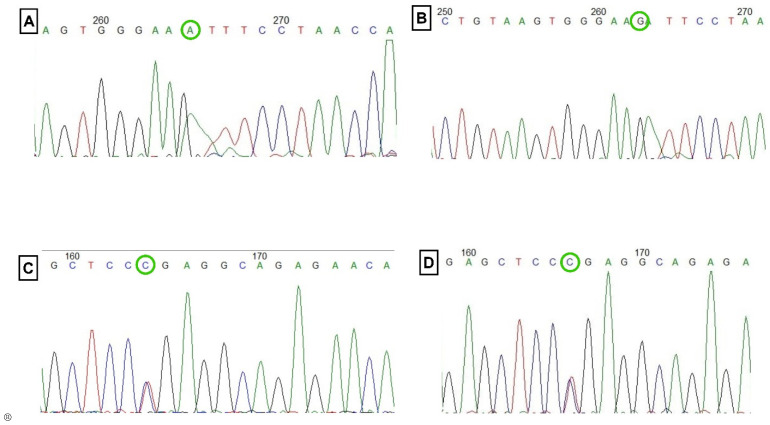
DNA Sequencing. (**A**,**B**) Direct DNA sequencing result for TNF-α rs1800629 polymorphism. (**C**) Direct DNA sequencing result for TGF-β rs1800469 polymorphism. (**D**) Direct DNA sequencing result for IL-1β rs16944 polymorphism.

One of the limitations of this study were that out of the three studied genes, TNF-α failed to comply with Hardy–Weinberg equilibrium. To validate the findings, Sanger sequencing in 10% of the samples was performed (Figure 2), which showed the same results as PCR-RFLP. This indicates that the results of TNF-α may not be applicable to the general population. However, this could also be due to the small sample size and the fact that the study only involves races of the North Indian population. Moreover, the study included subjects with ages restricted to thirty and above.

Additionally, we only studied three SNPs; however, SNPs at other loci may be more affected by the risk of CCS. Studies with a larger population and experimental studies exploring more in-depth molecular mechanisms are needed to establish these findings and to depict the detailed roles of these SNPs in the pathogenesis of CCS.

## 5. Conclusions

In conclusion, our findings demonstrated an association between three SNPs, IL-1β (-511C/T), TNF-α (-308C/T), and TGF-β (-509C/T), and the risk of the severity of chronic cervical spondylitis. Elevated serum levels of TNF-α and TGF-β also contribute to a better understanding of the disease’s molecular pathogenesis and may offer novel insights for the early detection and treatment of CCS. To better elucidate the functional roles of these polymorphisms in the etiology of CCS, in-depth molecular studies are required to clarify the biological signaling mechanisms involved in CCS in the North Indian population.

## Figures and Tables

**Table 1:** Primer sequences, PCR amplicon size, and restriction endonuclease of respective genes.

Genes	Primers Sequences	Amplicon Size (bp)	Restriction Enzyme (Product Sizes)
*IL-1β*			
Forward	5′-TGG CAT TGA TCT GGT TCA TC 3′	305	*AvaI* (190 + 115)
Reverse	5′-GGT TAG GAA TCT TCC CAC TT 3′		
*TNF-α*			
Forward	5′-AGG CAA TAG GTT TTGAGG GCC AT 3′	107	*NcoI* (87 + 20)
Reverse	5′-TCC TCC CTG CTC CGA TTC CG 3′		
*TGF-β*			
Forward	5′-GAG CAAT TCT TAC AGG TGT CTGC-3′	81	*Eco81I* (42 + 39)
Reverse	5′-GAG GGT GTC AGT GGG AGG AG-3′		

**Table 2:** Presentation of demographic data.

Characteristic	CCS (*n* = 126), f (%)	Control (*n* = 126), f (%)	*p*-Value
**Gender ratio (M/F)**	58(46.03%)/68(53.97%)	73(57.94%)/53(43.06%)	0.129
**Male (M)**	58 (44.3%)	73 (55.7%)	
**Female (F)**	68 (56.2%)	53 (43.8%)	
**Age (years) Mean ± SD**	47.23 ± 10.89	44.97 ± 11.32	0.097
**Height (cm) Mean ± SD**	157.87 ± 7.99	156.01 ± 8.25	0.07
**Smoker**	29 (23%)	31 (24.6%)	0.179
**ESR (mm/h) Mean ± SD**	40.056 ± 12.53	13.82 ± 4.75	0.0001 ***
**CRP (mg/L) Mean± SD**	-	1.68 ± 1.69	**-**

Descriptions: CCS = Chronic Cervical Spondylitis; SD = Standard Deviation; *n* = number; f = frequency; ESR = Erythrocyte Sedimentation Rate; CRP = C-reactive protein; cm = centimeter; mm/h = millimets/hour; *** highly significant; the *p*-value was calculated using the Chi square test.

**Table 3:** Genotypic and allelic distributions among CCS and control.

Genes	Genotypes	CCS, *n* (%)	Control, *n* (%)	OR (95% CI)	*p*-Value	Adjusted *p*-Value
*IL-1β (-511C/T)* (rs16944)	T/T	38 (30.16%)	25 (19.84%)		0.098	-
	C/C	26 (20.63%)	37 (29.37%)			
	C/T	62 (49.21%)	64 (50.79)			
	T/T	0.30 (38)	0.2 (25)	1 (Reference)		
	C/C	0.21 (26)	0.29 (37)	0.46 (0.23–0.94)	0.049 *	0.09
	C/T	0.49 (62)	0.51 (64)	0.64 (0.34–1.18)	0.166	-
Allele	T	0.55 (138)	0.45 (114)	1 (Reference)	0.04 *	0.08
	C	0.45 (114)	0.55 (138)	0.68 (0.48–0.97)		
*TNF-α (-308 A/G)* (rs1800629)	A/A	2 (1.59%)	1 (0.8%)		<0.0001 ***	<0.0003 ***
	G/G	26 (20.63%)	113 (89.68%)			
	A/G	98 (77.78%)	12 (9.52%)			
	A/A	0.02 (2)	0.01 (1)	1 (Reference)		
	G/G	0.2 (26)	0.89 (113)	0.11 (0.01–1.32)	0.1	-
	A/G	0.78 (98)	0.1 (12)	4.08 (0.34–48.5)	0.3	-
Allele	A	0.4 (102)	0.06 (14)	1 (Reference)	<0.0001 ***	<0.0002 ***
	G	0.6 (150)	0.94 (238)	0.87 (0.05–0.16)		
*TGF-β (-509C/T)* (rs1800469)	T/T	11 (8.73%)	5 (3.97%)		0.0039 **	0.023 *
	C/C	8 (6.35%)	0			
	T/C	107 (84.92%)	121 (96.03%)			
	T/T	0.09 (11)	0.04 (5)	1 (Reference)		
	C/C	0.06 (8)	0	8.13 (0.4–168)	0.13	-
	T/C	0.85 (107)	0.96 (121)	0.4 (0.14–1.2)	0.12	-
Allele	T	0.51 (129)	0.52 (131)	1 (Reference)	0.9	-
	C	0.49 (123)	0.48 (121)	1.03 (0.73–1.46)		

Descriptions: CCS = Chronic Cervical Spondylitis; *IL-1β* = Interleukin-1*β*; *TNF-α* = Tumor Necrosis Factor-*α*; *TGF-β* = Transforming Growth Factor-*β*; OR = Odd ratio; CI = Confidence Interval; * levels of significance (* *p* < 0.05; ** *p* < 0.01; *** *p* < 0.001); the *p*-value was calculated using the Chi square test, and Bonferroni correction was used for the adjusted *p*-value.

**Table 4:** Genotypic and Allelic distribution among different Age group.

	Age/Genotype	<50 Years, *n* (%)	>50 Years, *n* (%)	*p*-Value
***IL-1β***				
CCS	TT	26 (0.36)	12 (0.22)	0.234
	CC	13 (0.18)	13 (0.24)	
	CT	33 (0.46)	29 (0.54)	
	T-Allele	85 (0.59)	53 (0.49)	0.126
	C-Allele	59 (0.41)	55 (0.51)	
Control	TT	17 (0.18)	8 (0.24)	0.748
	CC	29 (0.31)	9 (0.27)	
	CT	47 (0.51)	16 (0.49)	
	T-Allele	81 (0.44)	32 (0.48)	0.56
	C-Allele	105 (0.56)	34 (0.52)	
***TNF-α***				
CCS	AA	1 (0.01)	1 (0.02)	0.8
	GG	15 (0.21)	11 (0.2)	
	AG	56 (0.78)	42 (0.78)	
	A-Allele	58 (0.4)	44 (0.41)	1
	G-Allele	86 (0.6)	64 (0.59)	
Control	AA	1 (0.01)	0	0.27
	GG	81 (0.87)	32 (0.97)	
	GA	11 (0.12)	1 (0.03)	
	A-Allele	13 (0.07)	1 (0.02)	0.12
	G-Allele	173 (0.93)	65 (0.98)	
***TGF-β***				
CCS	TT	7 (0.1)	4 (0.07)	0.67
	CC	4 (0.05)	5 (0.09)	
	CT	61 (0.85)	45 (0.84)	
	T-Allele	75 (0.52)	53 (0.49)	0.7
	C-Allele	69 (0.48)	55 (0.51)	
Control	TT	4 (0.04)	2 (0.06)	1
	CC	0	0	
	CT	89 (0.96)	31 (0.94)	
	T-Allele	97 (0.52)	35 (0.53)	1
	C-Allele	89 (0.48)	31 (0.47)	

Descriptions: CCS = Chronic Cervical Spondylitis; *IL-1β* = Interleukin-1*β*; *TNF-α* = Tumor Necrosis Factor-*α*; *TGF-β* = Transforming Growth Factor-*β*; OR = Odd ratio; CI = Confidence Interval; the *p*-value was calculated using the Chi square test.

**Table 5:** Genotypic distribution according to chronicity.

Chronicity/Genotype	>3 Months, *n* (%)	>1 Year, *n* (%)	*p*-Value
***IL-1β***			
TT	20 (0.34)	18 (0.27)	0.073
CC	7 (0.12)	19 (0.28)	
CT	32 (0.54)	30 (0.45)	
T-Allele	72 (0.61)	66 (0.49)	0.076
C-Allele	46 (0.39)	68 (0.51)	
***TNF-α***			
AA	0	2 (0.03)	0.34
GG	11 (0.19)	15 (0.22)	
AG	48 (0.81)	50 (0.75)	
A-Allele	48 (0.41)	54 (0.4)	1
G-Allele	70 (0.59)	80 (0.6)	
***TGF-β***			
TT	2 (0.03)	9 (0.13)	0.077
CC	6 (0.1)	3 (0.05)	
CT	51 (0.87)	55 (0.82)	
T-Allele	55 (0.47)	73 (0.54)	0.25
C-Allele	63 (0.53)	61 (0.46)	

**Table 6:** Genotypic distribution according to smokers.

Smoker/Genotype	CCS			Control		
	Non-Smoker, *n* (%)	Smoker, *n* (%)	*p*-Value	Non-Smoker, *n* (%)	Smoker, *n* (%)	*p*-Value
***Il-1β***						
TT	32 (0.31)	6 (0.26)	0.739	21 (0.19)	4 (0.24)	0.796
CC	22 (0.21)	4 (0.17)		34 (0.31)	4 (0.24)	
CT	49 (0.48)	13 (0.57)		54 (0.5)	9 (0.52)	
T-Allele	113 (0.55)	25 (0.54)	0.95	96 (0.44)	17 (0.5)	0.58
C-Allele	93 (0.45)	21 (0.46)		122 (0.56)	17 (0.5)	
***TNF-α***						
AA	2 (0.02)	0	0.368	1 (0.01)	0	0.876
GG	19 (0.18)	7 (0.3)		98 (0.9)	15 (0.88)	
AG	82 (0.8)	16 (0.7)		10 (0.09)	2 (0.12)	
A-Allele	86 (0.42)	16 (0.35)	0.41	12 (0.06)	2 (0.06)	1.0
G-Allele	120 (0.58)	30 (0.65)		206 (0.94)	32 (0.94)	
***TGF-β***						
TT	10 (0.1)	1 (0.04)	0.368	5 (0.05)	1 (0.06)	0.816
CC	6 (0.06)	3 (0.13)		0	0	
CT	87 (0.84)	19 (0.83)		104 (0.95)	16 (0.94)	
T-Allele	107 (0.52)	21 (0.46)	0.5	114 (0.52)	18 (0.53)	1.0
C-Allele	99 (0.48)	25 (0.54)		104 (0.48)	16 (0.47)	

**Table 7:** Genotypic and allelic distribution according to Smokers (*n* = 40) and non-smokers (*n* = 212) from CCS and control.

Smoker/Genotype	CCS Smoker, *n* (%)	Control Smoker, *n* (%)	*p*-Value	Adjusted *p*-Value
***Il-1β***				
TT	6 (0.26)	4 (0.24)	0.9	-
CC	4 (0.17)	4 (0.24)		
CT	13 (0.57)	9 (0.52)		
T-Allele	25 (0.54)	17 (0.5)	0.8	-
C-Allele	21 (0.46)	17 (0.5)		
***TNF-α***				
AA	0	0	0.001 **	0.003 **
GG	7 (0.3)	15 (0.88)		
AG	16 (0.7)	2 (0.12)		
A-Allele	16 (0.35)	2 (0.06)	0.003 ** (OR = 8.5)	0.006 *
G-Allele	30 (0.65)	32 (0.94)		
***TGF-β***				
TT	1 (0.04)	1 (0.06)	0.3	-
CC	3 (0.13)	0		
CT	19 (0.83)	16 (0.94)		
T-Allele	21 (0.46)	18 (0.53)	0.65	-
C-Allele	25 (0.54)	16 (0.47)		
	**CCS Non-Smoker,** ***n* (%)**	**Control Non-Smoker,** ***n* (%)**		
***Il-1β***				
TT	32 (0.31)	21 (0.19)	0.08	-
CC	22 (0.21)	34 (0.31)		
CT	49 (0.48)	54 (0.5)		
T-Allele	113 (0.55)	96 (0.44)	0.03 * (OR = 1.5)	0.06
C-Allele	93 (0.45)	122 (0.56)		
***TNF-α***				
AA	2 (0.02)	1 (0.01)	0.001 **	0.003 **
GG	19 (0.18)	98 (0.9)		
AG	82 (0.8)	10 (0.09)		
A-Allele	86 (0.42)	12 (0.06)	0.001 ** (OR = 12)	0.002 **
G-Allele	120 (0.58)	206 (0.94)		
***TGF-β***				
TT	10 (0.1)	5 (0.05)	0.01 *	0.02 *
CC	6 (0.06)	0		
CT	87 (0.84)	104 (0.95)		
T-Allele	107 (0.52)	114 (0.52)	1	-
C-Allele	99 (0.48)	104 (0.48)		

Descriptions: CCS = Chronic Cervical Spondylitis; *IL-1β* = Interleukin-1*β*; *TNF-α* = Tumor Necrosis Factor-*α*; *TGF-β* = Transforming Growth Factor-*β*; OR = Odd ratio; * levels of significance (* *p* < 0.05; ** *p* < 0.01); the *p*-value was calculated using the Chi square test.

**Table 8:** Correlation of serum cytokine levels in CCS and controls and their genotypes.

Cytokines	CCS, Median (Range)	Controls, Median (Range)	*p*-Value	*p*-Value
IL-1β (pg/mL)	4.4 (0.94–71.1)	3.6 (0.94–36.83)	0.336	0.001 **
TNF-α (pg/mL)	7.7 (0.38–129.78)	2.95 (0.38–94.8)	0.012 *	
TGF-β (pg/mL)	54.8 (18.7–614.3)	18.74 (18.74–531.4)	0.001 **	
Cytokines IL-1β (pg/mL)	Genotypes			*p*-value
	TT	CC	TC	
CCS	5.1 (0.94–53.2)	5.6 (0.94–71.1)	3.51 (0.94–28.2)	0.8
Control	2.9 (0.94–20.37)	3.8 (0.94–18.7)	3.8 (0.94–36.8)	
TNF-α (pg/mL)	AA	GG	AG	
CCS	22.56 (8.36–36.7)	7 (0.38–36.4)	7.2 (0.38–129.8)	0.01 *
Control	0.38	2.5 (0.38–94.8)	6.34 (0.38–31.1)	
TGF-β (pg/mL)	TT	CC	TC	
CCS	77.5 (18.7–389.6)	54.04 (18.7–149.4)	48.98 (18.7–614.3)	0.0001 *
Control	0	25.6 (18.7–107.1)	18.7 (18.74–531.4)	

Descriptions: CCS = Chronic Cervical Spondylitis; IL-1β = Serum Interleukin-1*β*; TNF-α = Serum Tumor Necrosis Factor-*α*; TGF-β = Serum Transforming Growth Factor- *β*; Serum Levels represented in Median (Range); * levels of significance (* *p* < 0.05; ** *p* < 0.01); the *p*-value was calculated using the Chi square test.

**Table 9:** Correlation of serum cytokine levels according to chronicity.

Cytokines/Chronicity	>3 Months, Median (Range)	>1 Year, Median (Range)	*p*-Value
IL-1β (pg/mL)	4.4 (0.94–53.2)	4.3 (0.94–71.1)	0.48
TNF-α (pg/mL)	5.46 (0.38–108.13)	10.38 (0.38–129.8)	0.23
TGF-β (pg/mL)	53.64 (18.74–391.54)	65.26 (18.74–614.3)	0.22

Descriptions: CCS = Chronic Cervical Spondylitis; IL-1β = Serum Interleukin-1*β*; TNF-α = Serum Tumor Necrosis Factor-*α*; TGF-β = Serum Transforming Growth Factor*-β*; Serum Levels represented in Median (Range); the *p*-value was calculated by the Chi square test.

**Table 10:** Correlation of serum cytokine levels in CCS and controls according to different age group.

Cytokines/Age	<50 Years, Median (Range)	>50 Years, Median (Range)	*p*-Value
**IL-1β** (pg/mL)			
CCS	3.4 (0.94–53.2)	4.6 (0.94–71.1)	1
Control	3.17 (0.94–28.16)	4.21 (0.94–36.8)	
**TNF-α** (pg/mL)			
CCS	3.4 (0.38–83.5)	8.65 (0.38–129.8)	0.34
Control	3.26 (0.38–80.16)	2.95 (0.38–94.8)	
**TGF-β** (pg/mL)			
CCS	80.5 (18.74–391.54)	54.04 (18.7–614.3)	0.35
Control	18.74 (18.74–337.89)	18.74 (18.74–531.42)	

Descriptions: CCS = Chronic Cervical Spondylitis; IL-1β = Serum Interleukin-1*β*; TNF-α = Serum Tumor Necrosis Factor-*α*; TGF-β = Serum Transforming Growth Factor-*β*; Serum Levels represented in Median (Range); the *p*-value was calculated by the Chi square test.

**Table 11:** Correlation of serum cytokine levels according to smokers.

Cytokines/Smoker	CCS, Median (Range)			Control, Median (Range)		
	Non-Smoker	Smoker	*p*-Value	Non-Smoker	Smoker	*p*-Value
**Il-1β** (pg/mL)	4.25 (0.94–53.2)	6.02 (0.94–71.1)	0.31	3.77 (0.94–36.8)	2.87 (0.94–20.2)	0.12
**TNF-α** (pg/mL)	8.5 (0.38–129.78)	4.26 (0.38–48.4)	0.37	2.03 (0.38–80.2)	8.7 (0.38–94.8)	0.02 *
**TGF-β** (pg/mL)	77.75 (18.74–614.3)	44.67 (18.74–232.6)	0.59	18.74 (18.74–337.9)	25.27 (18.74–531.42)	0.3

Descriptions: CCS = Chronic Cervical Spondylitis; IL-1β = Serum Interleukin-1*β*; TNF-α = Serum Tumor Necrosis Factor-*α*; TGF-β = Serum Transforming Growth Factor-*β*; Serum Levels represented in Median (Range); * levels of significance; the *p*-value was calculated using the Chi square test.

**Table 12:** Correlation study of variables with genotypes.

Variables		Chronicity (Pc/*p* < 0.05)	*IL-1β* (Pc/*p* < 0.05)		*TNF-α* (Pc/*p* < 0.05)		*TGF-β* (Pc/*p* < 0.05)	
Genotype Frequency		CCS	CCS	Control	CCS	Control	CCS	Control
*Age*	CCS	0.304/0.001 *	0.058/0.52	−0.014/0.87	−0.065/0.47	−0.083/0.35	−0.08/0.36	0.07/0.43
	Control	0.066/0.46	0.07/0.41	−0.108/0.23	0.044/0.62	−0.125/0.16	−0.063/0.48	−0.008/0.93
*Height*	CCS	0.05/0.55	0.007/0.94	−0.04/0.64	0.031/0.73	−0.076/0.34	0.034/0.7	−0.18/0.044
	Control	0.06/0.49	0.046/0.61	0.01/0.91	−0.19/0.026 *	0.057/0.53	0.12/0.19	0/0.99
*ESR*	CCS	−0.05/0.58	−0.03/0.73	−0.054/0.54	0.09/0.31	0.01/0.9	−0.204/0.02 *	−0.17/0.052
	Control	0.14/0.102	0.023/0.8	−0.15/0.089	−0.013/0.13	−0.12/0.19	−0.62/0.49	0.005/0.95
*IL-1β*	CCS	−0.015/0.86	1	−0.038/0.67	0.13/0.14	−0.062/0.49	−0.06/0.49	−0.037/0.68
	Control	−0.109/0.223	−0.038/0.67	1	0.2/0.025 *	0.22/0.013 *	−0.03/0.75	−0.009/0.92
*TNF-α*	CCS	−0.09/0.31	0.13/0.14	0.2/0.025 *	1	0.146/0.1	−0.125/0.16	−0.035/0.7
	Control	−0.087/0.33	−0.06/0.49	0.22/0.013 *	0.146/0.104	1	−0.013/0.89	−0.057/0.52
*TGF-β*	CCS	−0.13/0.14	−0.06/0.49	−0.028/0.75	−0.125/0.16	−0.013//0.89	1	−0.09/0.3
	Control	0.107/0.23	−0.037/0.68	−0.009/0.92	−0.035/0.7	−0.057/0.52	−0.09/0.307	1

Descriptions: Pc = Pearson Correlation; CCS = Chronic Cervical Spondylitis; *IL-1β* = Interleukin-1*β gene*; *TNF-α* = Tumor Necrosis Factor-*α gene*; *TGF-β* = Transforming Growth Factor-*β gene*; ESR = Erythrocyte Sedimentation Rate; * Levels of significance (* *p* < 0.05); the *p*-value was calculated using a Chi square test.

**Table 13:** Correlation study of serum cytokines with genotype frequency.

Variables		Chronicity (Pc/*p* < 0.05)	*IL-1β* (Pc/*p* < 0.05)		*TNF-α* (Pc/*p* < 0.05)		*TGF-β* (Pc/*p* < 0.05)	
Levels/Frequency		CCS	CCS	Control	CCS	Control	CCS	Control
IL-1β	CCS	−0.009/0.93	−0.12/0.236	−0.006/0.95	−0.014/0.89	−0.087/0.4	0.046/0.65	−0.014/0.89
	Control	-	−0.02/0.86	0.076/0.45	−0.23/0.02 *	0.012/0.9	−0.07/0.48	0.094/0.35
TNF-α	CCS	0.175/0.08	0.13/0.186	0.04/0.69	0.056/0.58	−0.06/0.55	0.065/0.5	−0.02/0.84
	Control	-	−0.04/0.67	0.04/0.68	0.075/0.45	−0.014/0.89	−0.06/0.52	−0.21/0.36
TGF-β	CCS	0.097/0.34	0.058/0.57	0.1/0.29	−0.12/0.24	−0.15/0.13	−0.048/0.64	0.1/0.315
	Control	-	−0.03/0.77	0.19/0.06	−0.012/0.9	0.017/0.86	−0.08/0.41	0.035/0.728

Descriptions: Pc = Pearson Correlation; CCS = Chronic Cervical Spondylitis; *IL-1β* = Interleukin-1*β gene*; *TNF-α* = Tumor Necrosis Factor-*α gene*; *TGF-β* = Transforming Growth Factor-*β gene*; IL-1β, TNF-α, TGF-β = Serum Cytokines; * Levels of significance; the *p*-value was calculated using a Chi square test.

## Data Availability

As requested.

## Supplementary Materials

The following supporting information can be downloaded at https://www.mdpi.com/article/10.3390/cells12121594/s1. Supplementary Figure S1: Agarose gel image of IL-1β; Supplementary Figure S2: Agarose gel image of TNF-α; Supplementary Figure S3: Native PAGE image of TGF-β.

## Abbreviations

CCS	Chronic cervical spondylitis
SNPs	Single-nucleotide polymorphisms
IL-1β	Interlaken 1 beta
TNF-α	Tumor necrosis factor-alfa
TGF-β	Tumor necrosis factor-beta
PAGE	Polyacrylamide gel electrophoresis
RFLP	Restriction Fragment Length Polymorphism
EDTA	Ethylenediamine tetraacetic acid
ESR	Erythrocyte Sedimentation Rate
CRP	C-reactive Protein
OA	Osteoarthritis
AS	Ankylosing spondylitis
RA	Rheumatoid arthritis
BMI	Body Mass Index
VDR	Vitamin-D Receptor
MRI	Magnetic resonance imaging
AP1	Activated protein 1
