# Supplementary material for: Genetic Variations in IL-1β, TNF-α, and TGF-β Associated with the Severity of Chronic Cervical Spondylitis in Patients
Source: Cells. 2023 Jun 9;12(12):1594. doi: 10.3390/cells12121594 (PMC10297355; doi:10.3390/cells12121594)
Supplement: Supplementary file 1 [file cells-12-01594-s001.zip › cells-2380366-supplementary.PPTX]

## Slide 1
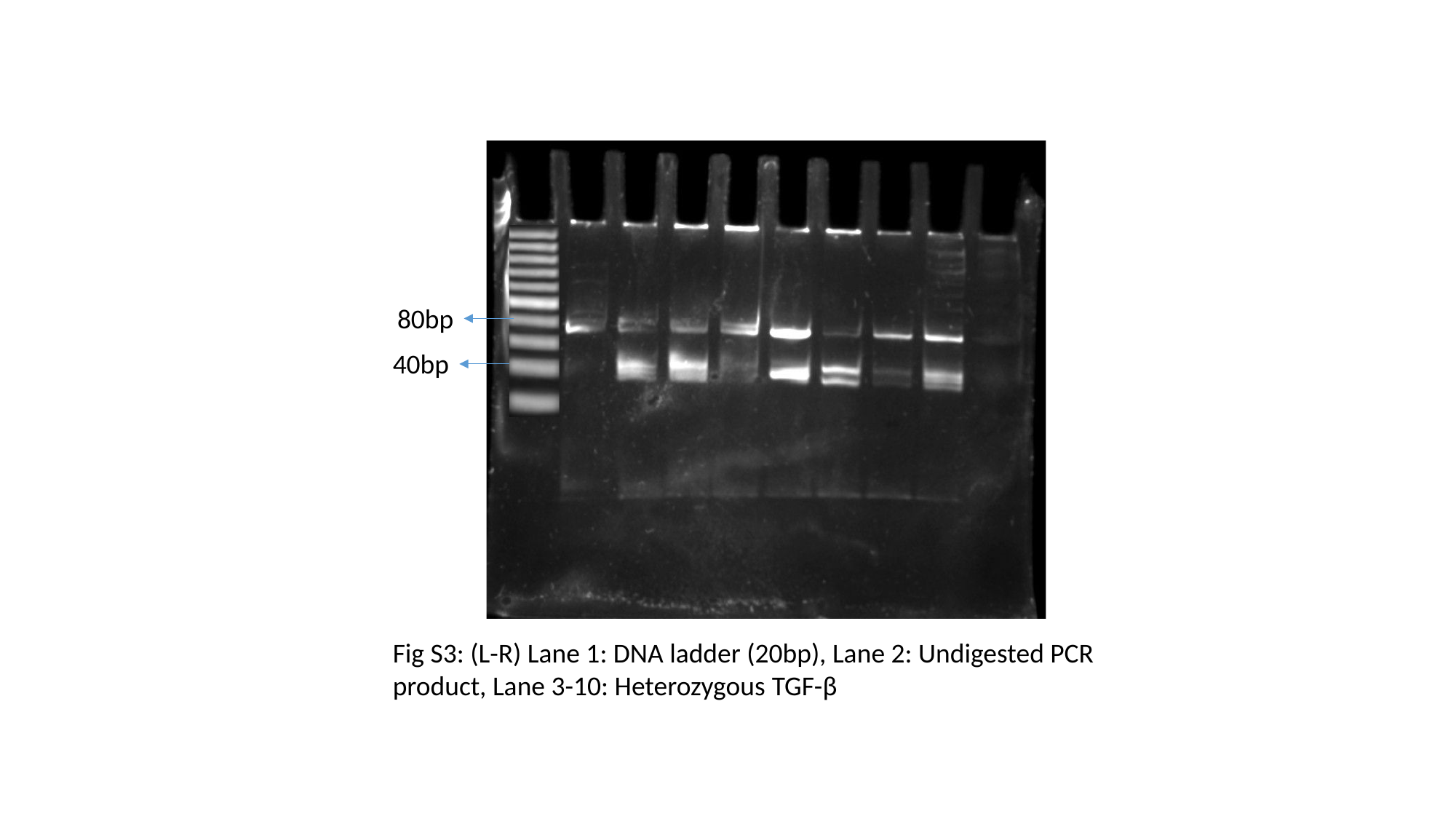

80bp
40bp
Fig S3: (L-R) Lane 1: DNA ladder (20bp), Lane 2: Undigested PCR product, Lane 3-10: Heterozygous TGF-β

## Slide 2
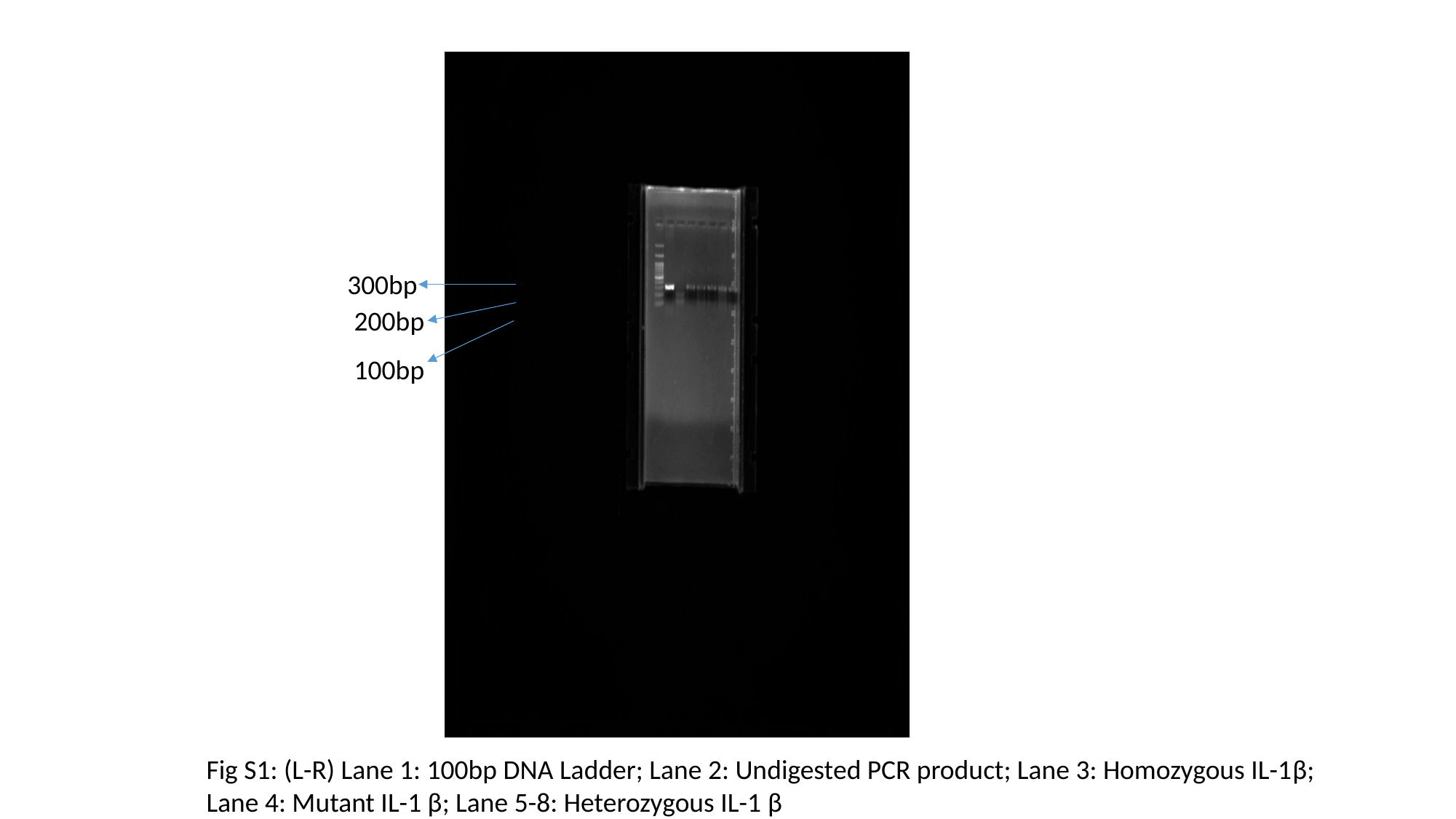

300bp
200bp
100bp
Fig S1: (L-R) Lane 1: 100bp DNA Ladder; Lane 2: Undigested PCR product; Lane 3: Homozygous IL-1β; Lane 4: Mutant IL-1 β; Lane 5-8: Heterozygous IL-1 β

## Slide 3
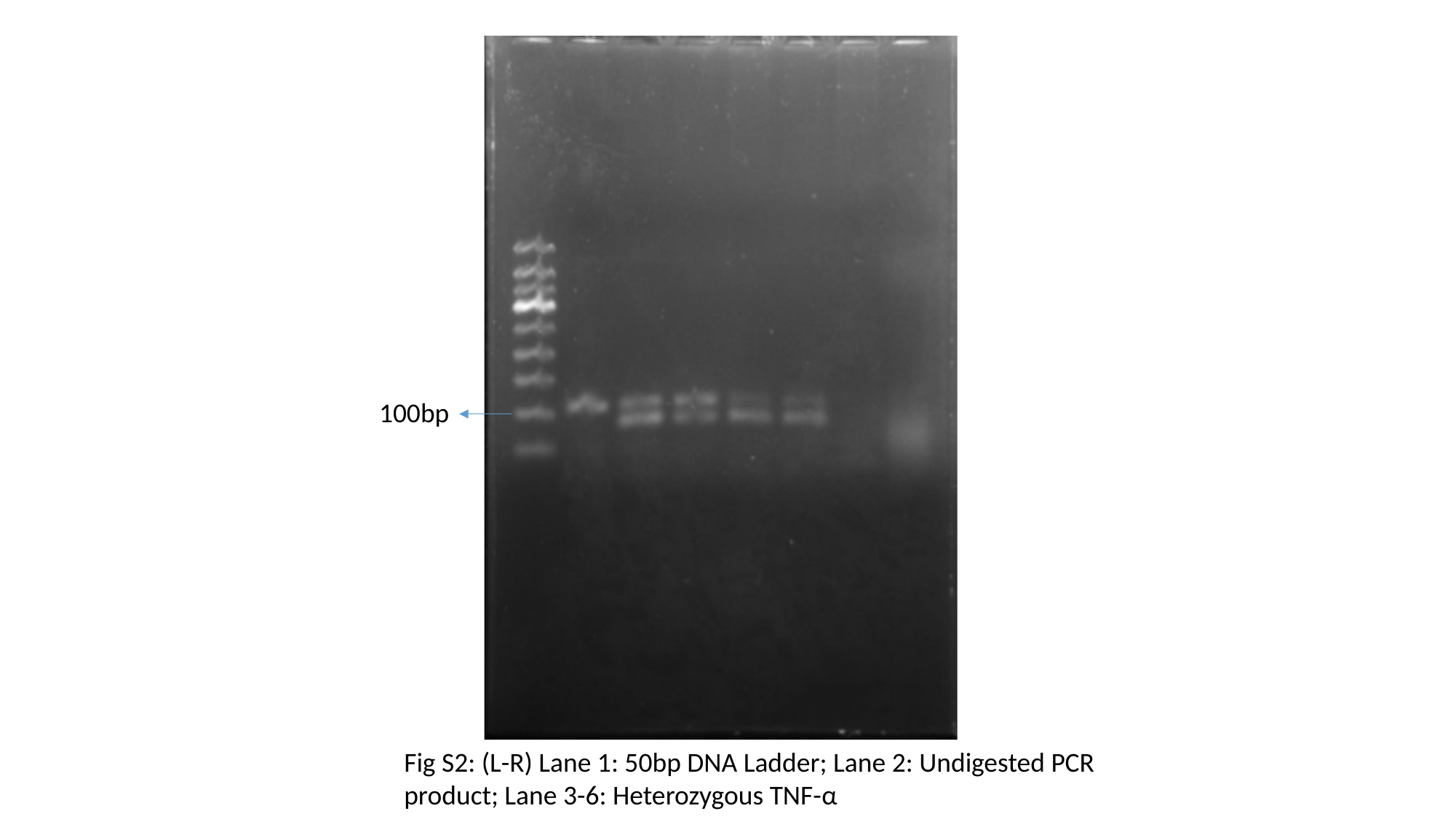

100bp
Fig S2: (L-R) Lane 1: 50bp DNA Ladder; Lane 2: Undigested PCR product; Lane 3-6: Heterozygous TNF-α
